# Timing and Dose of Constraint-Induced Movement Therapy after Stroke: A Systematic Review and Meta-Regression

**DOI:** 10.3390/jcm12062267

**Published:** 2023-03-15

**Authors:** Yu-Kai Yang, Chieh-Yu Lin, Po-Huang Chen, Hong-Jie Jhou

**Affiliations:** 1 Department of Physical Medicine and Rehabilitation, Changhua Christian Hospital, Changhua 500209, Taiwan; hermiterudite@gmail.com; 2 Division of General Practice, Department of Medical Education, Changhua Christian Hospital, Changhua 500209, Taiwan; channy8411@gmail.com; 3 Department of Internal Medicine, Tri-Service General Hospital, National Defense Medical Center, Taipei 114, Taiwan; 4 Department of Neurology, Changhua Christian Hospital, Changhua 500209, Taiwan; 5 School of Medicine, Kaohsiung Medical University, Kaohsiung 807, Taiwan

**Keywords:** constraint-induced movement therapy (CIMT), stroke, cognitive function, moto activity log, Fugl-Meyer assessment, wolf motor function test

## Abstract

The aim of this study is to investigate the effects of constraint-induced movement therapy on stroke patients who had intact cognition and some voluntary finger extension and to identify optimal protocols to apply this therapy method. We searched PubMed, Cochrane Library, and Embase for randomized controlled trials conducted prior to January 2022. The outcomes included the Motor Activity Log, Fugl-Meyer Assessment, and Wolf Motor Function Test. The inverse variance method fixed-effect model as well as the DerSimonian and Laird estimator random-effects model were applied, and the mean difference was calculated with 95% confidence interval to measure continuous outcomes. Six randomized controlled trials involving a total of 169 patients with stroke were enrolled. Compared with conventional rehabilitation methods, there was no significant effect of constraint-induced movement therapy when evaluated by the Motor Activity Log, including the amount of use (random-effect, standardized mean difference 0.65; 95%, confidence interval: −0.23–1.52) and quality of movement (random-effect, standardized mean difference 0.60; 95% confidence interval: −0.19–1.39). However, among patients with chronic stroke symptoms, meta-regression analyses showed better performance with a constraint time of at least 6 h per day and 6 h training per week when assessing the amount of use (*p* = 0.0035) and quality of movement (*p* = 0.0031). Daily intervention time did not lead to a significant difference in functional upper limb performance. An efficient protocol of constraint-induced movement therapy designed as 6 h of training per week with 6 h constraint per day could bring significant stroke symptom improvement to patients with chronic stroke.

## 1. Introduction

Stroke is the third most common cause of disability and the second most common cause of death worldwide [1,2]. There are 101 million people worldwide living with post-stroke symptoms, and this number has almost doubled over the last 30 years. Additionally, the lifetime prevalence of stroke has increased 50% over the last 17 years. Up to 88% of patients diagnosed with an acute stroke experience hemiparesis [3]. It is typical for hemiparesis patients to experience a greater degree of paretic effect in the arm versus the leg, and the degree of functional motor recovery in the arm is less than in that of the leg. Previous studies of patients with hemiparesis show that early control of finger extension is a crucial prognostic indicator for the recovery of upper extremity dexterity [4,5].

Constraint-induced movement therapy is designed to improve upper extremity motor function after stroke, and it is especially recommended for those with adequate activation. Constraint-induced movement therapy involves restraint of the ipsilesional limb, which means a less affected limb, as well as repetitive task-oriented practice and behavioral shaping techniques in the more affected limb. Several studies demonstrate the efficacy of constraint-induced movement therapy in patients with acute (1–14 days poststroke) [6], subacute (<6 months post-stroke) [7], and chronic stroke (beyond 6 months) [8,9,10]. However, other studies show negative results of constraint-induced movement therapy [11,12]. The discrepancies in research conclusions may result from variation between study designs, including the duration and frequency of treatment, constraint time, and the time between stroke occurrence and constraint-induced movement therapy trial enrollment. In addition, patients have low treatment adherence due to long constraint time.

Previous systematic reviews have put emphasis on assessing the effects of constraint-induced movement therapy among patients with chronic, [13,14] acute, and subacute stroke [7,15]. Consequently, a new systematic review that proposes clear timing and protocol of constraint-induced movement therapy is crucial.

The goal of the present meta-analysis is to explore the efficacy of constraint-induced movement therapy among stroke survivors with fair cognitive function who retain the ability to actively extend the fingers and wrist of their paretic upper extremity. This meta-analysis also attempts to establish an efficient protocol to improve clinical implementation of constraint-induced movement therapy.

## 2. Materials and Methods

This report is a systematic review and meta-analysis of randomized controlled trials according to the guideline of Preferred Reporting Items for Systematic Reviews and Meta Analyses [16] (Table S1). The review protocol was registered with the Open Science Framework platform, available at https://osf.io/uprtk (accessed on 10 February 2022). All data supporting the outcomes of this study are available from the corresponding author upon reasonable request.

### 2.1. Data Sources and Search Strategy

Relevant research was systemically identified through PubMed, Embase, and the Cochrane Library through a review of study titles and abstracts. Conference abstracts and reference lists of included studies were comprehensively reviewed to ensure that no randomized controlled trials were overlooked.

The systematic search for randomized controlled trials was conducted by two independent reviewers. All studies prior to January 2022 were reviewed using the search terms “stroke”, “infarction”, “forced use”, “constraint-induced therapy”, and “constraint-induced movement therapy”. The two reviewers assessed full texts of the studies independently. Studies were included if they satisfied the predetermined criteria. Any disagreement between the two reviewers was resolved by consensus.

### 2.2. Study Selection

Inclusion criteria: (1) randomized control trials with at least two measurement points (including pre- and post-intervention outcome); (2) participants aged older than 18 years; (3) reported a single stroke event, whether ischemic or hemorrhagic; (4) participants with the ability to actively extend 10° at the wrist, metacarpophalangeal joint, and interphalangeal joints [17]; (5) participants without serious cognitive deficits, which were defined as above 23 points in the Mini Mental State Exam or 77 points in the modified Mini Mental State Exam [18,19]; (6) studies comparing constraint-induced movement therapy (or a modified form of constraint-induced movement therapy) to a control group receiving dose-matched traditional rehabilitation therapy; (7) studies that adopted at least one of the following assessment indicators: Fugl-Meyer assessment of the arm, the Motor Activity Log, or Wolf Motor Function Test; (8) studies published without language restrictions. Exclusion criteria: (1) studies including participants aged younger than 18 years; (2) studies including patients with severe upper extremity hemiparesis; (3) studies without an active comparison group (no treatment or placebo).

### 2.3. Data Extraction and Bias Assessment

The data from all included studies were independently extracted by two reviewers. Data extracted by the reviewers included the study title, year of publication, post-stroke duration, experimental and control numbers, mean age of participants, intervention time (including duration, frequency, and total training time), constraint time, original outcomes of individual trials, and any characteristics related to the study, participant, or management. The reviewers appraised the quality of the included randomized control trial studies by using the Cochrane Risk of Bias tool, [20] which assesses risk within six domains (selection bias, detection bias, performance bias, reporting bias, attrition bias, and other bias). The results were categorized as high, low, or unclear risk (Figure S1).

### 2.4. Outcome Measures

Data extracted from the studies included in this systematic review identified three subjective measurements of an individual’s upper limb functional ability in daily life, including Motor Activity Log [21], Fugl-Meyer assessment [22], and the Wolf Motor Function Test [23].

The Motor Activity Log is a semi-structured interview that evaluates patients in 30 important daily activities. A six-point amount of use scale (score range, 0–5) was designed to rate how often the affected arm was being used, and a six-point quality of movement scale was used to assess how well the affected limb was being used. According to the number of items, there were different versions of the original Motor Activity Log −30 [24].

The Fugl-Meyer assessment was designed for evaluating recovery from motor impairment, including movement, reflexes, coordination, and speed, via 33 upper limb assessment items. The score of each item was calculated with a three-point ordinal scale ranging from 0 (cannot perform) to 2 (performs fully). The reliability and construct validity of the Fugl-Meyer assessment are well established [25]. The Wolf Motor Function Test assesses changes in upper limb motor function through 15 functional tasks. Quality of movement scoring was assessed by a six-point functional ability scale, which ranged from 0 (not attempted) to 5 (normal movement) [26,27].

### 2.5. Statistical Analysis

The data were analyzed using the *Cochrane Handbook for Systematic Reviews of Interventions* [28]. The inverse variance method fixed-effect model as well as the DerSimonian and Laird estimator random-effects model were applied [29], and the mean difference was calculated with 95% confidence intervals to measure continuous outcomes [30]. We calculated the standardized mean difference with 95% confidence intervals for the Motor Activity Log because of the different measurement scales. Heterogeneity was assessed with the *I* square (*I*^2^) statistic [31] and Cochran’s Q test [32], where an *I*^2^ greater than 50% and Cochran’s Q test *p* < 0.1 indicated statistically significant heterogeneity.

Subgroup analysis was conducted by evaluating differences in post-stroke duration. Subgroups were divided into either chronic (>6 months) or subacute phase (<6 months) [33], and by length of constraint time (more or less than 3 h) [15]. The differences between these subgroups were examined across the various assessments involving the Motor Activity Log, including the amount of use and quality of movement scale, the functional ability scale of the Wolf Motor Function Test, and the Fugl-Meyer assessment. A mixed-effects linear meta-regression model [34] was used to analyze the cause of heterogeneity for all outcomes involving variables with post-stroke duration, intervention time, constraint time, training hours per week, total course, and total training time. Intention-to-treat analysis exploring attributional bias was performed.

This study conducted all statistical analyses by using “meta” packages of R software version 3.6.1. [35] and “metafor” [36]. Statistical significance was defined as a *p* value of <0.05.

## 3. Results

**Figure 1:**
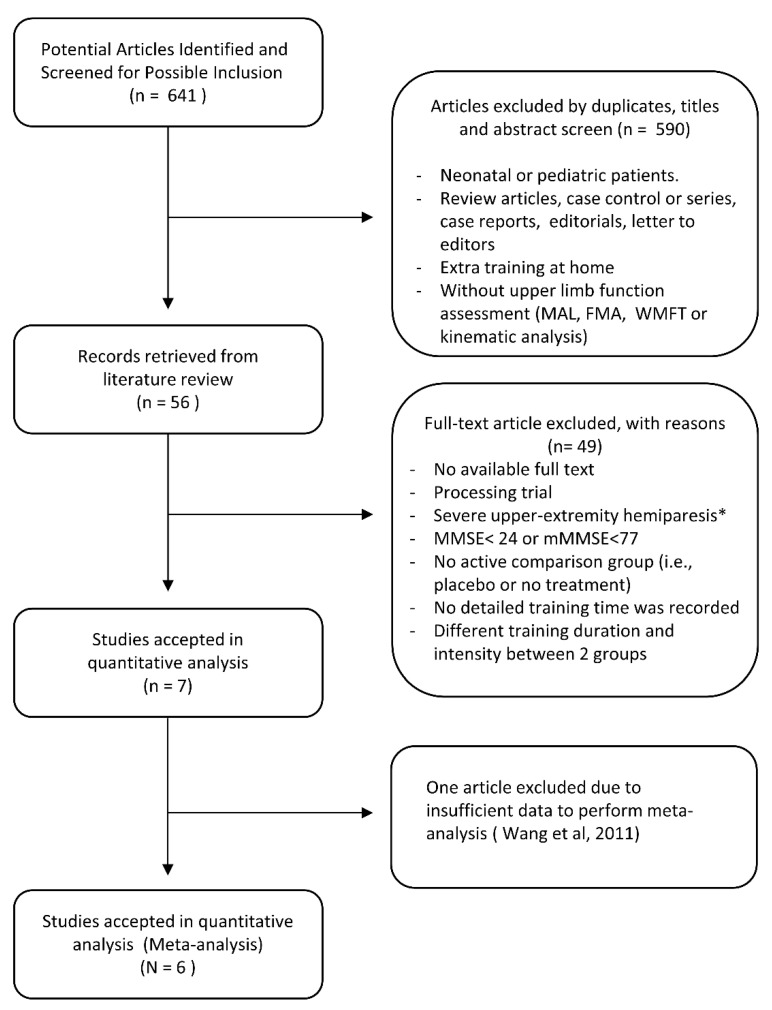
Flowchart of literature search process to obtain eligible trials. * At least 10° of active extension of wrist, metacarpophalangeal joint, and interphalangeal joint (Smania et al., 2012 [17], Wang et al., 2011 [37]).

A total of 641 potentially relevant studies were identified according to the initial literature search strategy. Subsequently, 590 of these studies were excluded based on reviewing the title and abstract, including exclusion of any duplicated articles. After full-text review, an additional 49 studies were excluded from the remaining 56 studies for varying reasons, such as studies that included participants exhibiting serious cognitive deficits, severe upper extremity hemiparesis, or studies including different training durations and intensities between two groups. One of the remaining seven studies was excluded due to insufficient data to perform meta-analysis [37]. In total, six randomized controlled trials were included in the final meta-analysis (Figure 1).

These six randomized control trials selected for the meta-analysis included a total of 169 patients categorized into two groups: 84 patients receiving constraint-induced movement therapy and 85 receiving traditional rehabilitation therapy. Patient characteristics of all included randomized trials are presented in Table 1. The included randomized controlled trials were published between 2010 and 2021. The sample sizes in the included studies ranged between 13 and 59 patients. The range of post-stroke duration was between 1.41 and 42.78 months (mean: 14.81 months). The effects of constraint-induced movement therapy were evaluated in five studies using the Motor Activity Log, in three studies using the Wolf Motor Function Test, and in three studies using the Fugl-Meyer assessment.

### 3.1. Outcomes

**Figure 2:**
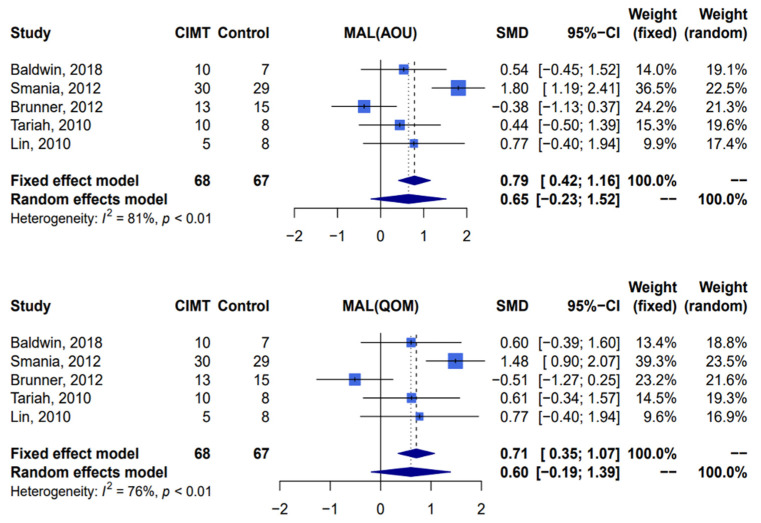
Meta-analysis of Motor Activity Log-amount of use and Motor Activity Log-quality of movement. CIMT, constraint-induced movement therapy; MAL, Motor Activity Log; AOU, amount of use; QOM, quality of movement; SMD, standardized mean difference; CI, confidence interval [11,17,38,39,40].

Five studies [11,17,38,39,40] with a total of 135 patients were included in the analysis. Compared with patients receiving traditional rehabilitation therapy, there was no significant effect on the functional ability among patients receiving constraint-induced movement therapy with the Motor Activity Log, including amount of use scale (random-effect, standardized mean difference 0.65; 95% confidence interval: −0.23–1.52; *I*^2^ = 81%, Cochran’s Q *p* < 0.01) and quality of movement scale (random-effect, standardized mean difference 0.60; 95% confidence interval: −0.19–1.39; *I*^2^ = 76%, Cochran’s Q *p* < 0.01) (Figure 2).

**Figure 3:**
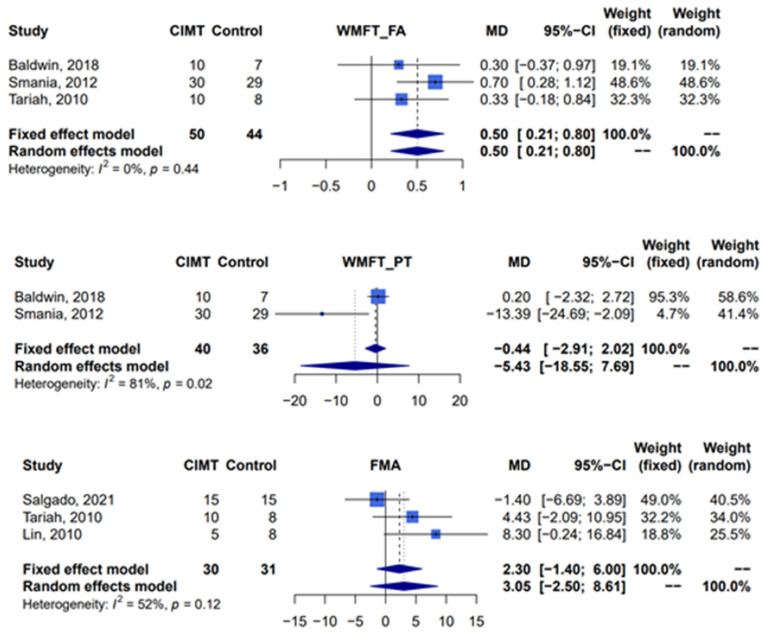
Meta-analysis of Wolf Motor Function Test and Fugl-Meyer assessment. CIMT, constraint-induced movement therapy; WMFT, Wolf Motor Function Test; FA, functional ability; PT, performance time; FMA, Fugl-Meyer assessment; MD, mean difference; CI, confidence interval [10,11,17,39,40].

Compared with traditional rehabilitation therapy, constraint-induced movement therapy was associated with a statistically significant effect when assessed by the functional ability scale of the Wolf Motor Function Test (three studies [11,17,39], *n* = 94 patients, random-effect, mean difference 0.5; 95% confidence interval: 0.21–0.80; *I*^2^ = 0%, Cochran’s Q *p* = 0.44). Due to one study lacking sufficient data for calculation [39], only two studies were included in the assessment of the effect of constraint-induced movement therapy by the performance time scale of the Wolf Motor Function Test, and this assessment showed that there was no significant difference between two groups (two studies [11,17], *n* = 76 patients, random-effect, mean difference −5.43; 95% confidence interval: −18.55–7.69; *I*^2^ = 81%, Cochran’s Q *p* = 0.02). Analysis of the Fugl-Meyer assessment, which was used to evaluate motor impairment, revealed that there was no significant difference between constraint-induced movement therapy and the control group (three studies [10,39,40], *n* = 61 patients, random-effect, mean difference 3.05; 95% confidence interval: −2.50–8.61; *I*^2^ = 52%, Cochran’s Q *p* = 0.12) (Figure 3).

### 3.2. Subgroup and Meta-Regression Analyses

Subgroup analysis was conducted for the different post-stroke duration and constraint time groups. In the subgroup of patients who suffered from chronic stroke symptoms (over 6 months’ duration), analysis showed a better effect on functional ability as evaluated by the Motor Activity Log and the Wolf Motor Function Test. The analysis details can be found in the supplementary data (Figure S2). We also found increased efficacy of the functional ability scale of the Wolf Motor Function Test for the subgroup of patients undergoing constraint for >3 h (random-effect; mean difference 0.59; 95% confidence interval: 0.23–0.94; *I*^2^ = 0%; *p* = 0.32).

**Figure 4:**
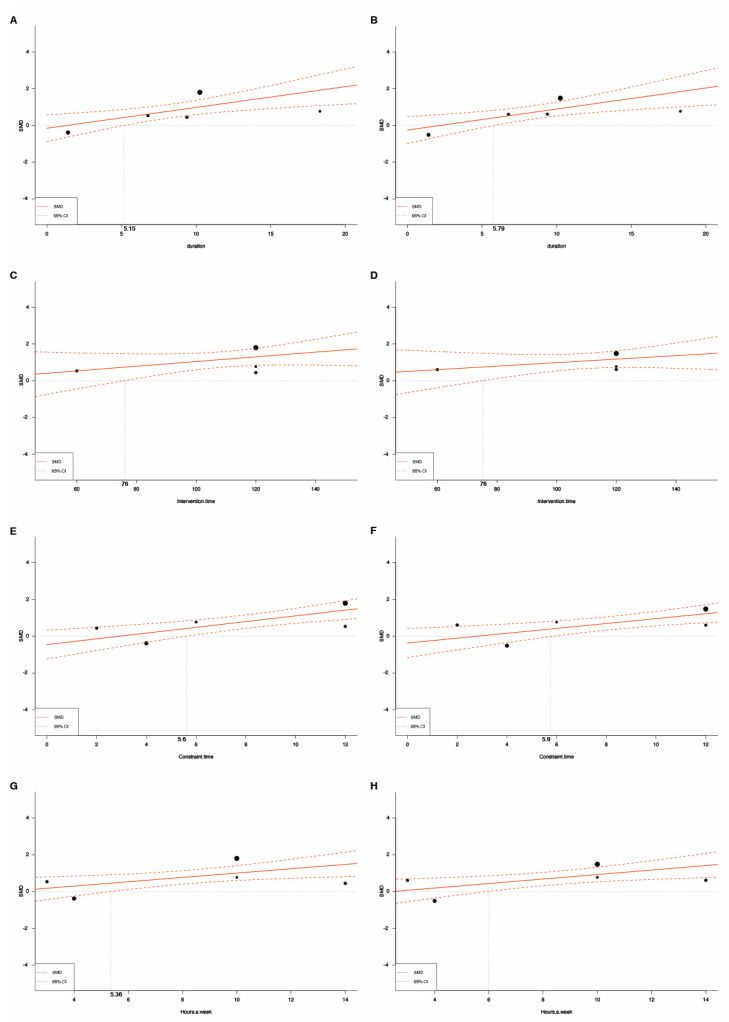
Meta-regression analysis: (**A**) evaluate the efficacy of constraint–induced movement therapy among patients with different post–stroke duration by using the amount of use scale of the Motor Activity Log; (**B**) evaluate the efficacy of constraint-induced movement therapy among patients with different post–stroke duration by using the quality of movement scale of the Motor Activity Log; (**C**) evaluate the efficacy of intervention time by using the amount of use scale of the Motor Activity Log; (**D**) evaluate the efficacy of intervention time by using the quality of movement scale of the Motor Activity Log; (**E**) evaluate the efficacy of constraint time by using the amount of use scale of the Motor Activity Log; (**F**) evaluate the efficacy of constraint time by using the quality of movement scale of the Motor Activity Log; (**G**) evaluate the efficacy of training hours per week by using the amount of use scale of the Motor Activity Log; (**H**) evaluate the efficacy of training hours per week by using the quality of movement scale of the Motor Activity Log.

Meta-regression analysis was conducted to examine the relationship between the following six variables: post-stroke duration, intervention time, constraint time, training hours per week, course, and total training time. Compared with the usual care, constraint-induced movement therapy increased functional ability on the Motor Activity Log, including the amount of use (five studies [11,17,38,39,40], coefficient 0.113 (0.037–0.189), *p* = 0.0035) and quality of movement (five studies [11,17,38,39,40], coefficient 0.115 (0.039–0.190), *p* = 0.0031) among patients with chronic stroke symptoms. Increased amount of use and quality of movement were also noted with increased constraint time of at least 6 h per day and 6 h of training per week (Table 2 and Figure 4). Due to one study [38] lacking details regarding intervention time, four studies were analyzed in this meta-regression of intervention time. However, daily intervention time did not lead to a significant difference in functional upper limb performance.

## 4. Discussion

This systematic review and meta-analysis investigated the efficacy of constraint-induced movement therapy in stroke patients with preserved upper limb activity and cognitive function. The available evidence suggests that constraint-induced movement therapy is more beneficial than traditional rehabilitation therapy for improvement of upper extremity functional ability (as defined by the functional ability scale of the Wolf Motor Function Test). The enhancement of activity outcome (as defined by the Motor Activity Log, including the quality of movement and the amount of use scales) was notably significant in patients with chronic stage stroke, which reflects the results of previous studies [41,42].

Previous systematic reviews have put emphasis on assessing the effect of constraint-induced movement therapy among post-stroke patients [7,13,14,43]. However, none of them mentioned efficient protocol. As far as we know, only one recent systematic review^1^ investigated dose, timing, and efficacy of upper limb rehabilitation during acute and subacute stage after stroke. However, it merely concluded that the median dose of intervention was [44] (interquartile range, 600–1430) min/session, 1 (interquartile range, 1–1) session/day, 5 (interquartile range, 5–5) day/week for 4 (interquartile range, 3–5) weeks. In addition, it did not focus on constraint-induced movement therapy. Consequently, a new systematic review proposing a clear timing and protocol of constraint-induced movement therapy is crucial. The current meta-analysis revealed that 6 h training per week with 6 h constraint per day is significantly effective.

For upper extremity recovery, 80% of patients achieved maximum recovery in three weeks and 95% achieved maximum recovery in nine weeks [45]. Constraint-induced movement therapy used in the chronic stroke symptom phase can improve motor impairment in the upper limb. Early intervention with intense rehabilitation programs is not recommended [46,47] because it may cause neuronal injury and impair synaptic plasticity later at the acute or subacute stages [44,45]. In the present meta-analysis, patients with chronic stage stroke experienced better improvement, which is consistent with prior studies. The results of this meta-regression suggest that patients should receive constraint-induced movement therapy at least 6 months after onset of stroke.

The constraint-induced movement therapy protocol proposed by Taub et al. is composed of 6 h of supervised training of the paretic limb daily and restriction of the non-paretic limb for 90% of the time. Therapy intensity and constraint time have been repeatedly described as limiting factors in the implementation of constraint-induced movement therapy or modified forms of constraint-induced movement therapy in standard care [13,48]. One hour of daily constraint-induced movement therapy three times per week without additional restriction time was sufficient to increase motor function of the upper limb and functional range in a recent trial [10]. Nevertheless, a potential dose effect was shown in our regression analysis. The difference may be connected to variations in the protocol and characteristics of participants. In former studies, all participants were right-handed and had experienced ischemic stroke with predominant right hemisphere lesion (56.7%). We determined that no less than 6 h of constraint per day was the most beneficial for patients.

The present outcomes did not include kinematic measures, such as movement duration and peak velocity, which were included in several prior research studies. Kinematic analysis provided more precise measurement in assessing the improvement through time and space. It also characterized the structure and quality of the movement, which assisted in the understanding of the underlying neural mechanisms [49,50,51]. Nevertheless, objective clinical outcome measures showed no inferiority to kinematic variables in responsiveness [50].

Several clinical trials are currently evaluating the effects of constraint-induced movement therapy used in combination with different technologies, including robotic therapy [52] and threshold electrical stimulation [53]. The findings of these studies may help to guide future studies design their inclusion criteria and protocol.

Our meta-analysis has demonstrated the effectiveness of constraint-induced movement therapy and established an efficient protocol. To minimize bias arising from different protocols, we used subgroup analysis and meta-regression. However, the evaluation of long-term efficacy of constraint-induced movement therapy was not possible due to heterogeneity in the follow-up period, courses of therapy, and recruitment period (months after stroke) across the included studies, which may have affected the reliability of the results. In addition, incomplete recording of some outcomes of interest in the randomized controlled trials hindered comprehensive subgroup analysis. Furthermore, we encountered a paucity of large clinical trials meeting our inclusion criteria to assess the efficacy of constraint-induced movement therapy, particularly those with kinematic measures [9,51,54,55]. Despite these limitations, we believe our analysis provides valuable insights into the efficacy of constraint-induced movement therapy for stroke patients and may guide the development of more standardized protocols for future studies in this area. Finally, we implemented a set of rigorous inclusion criteria to ensure homogeneity in our meta-analysis given the various protocols for constraint-induced movement therapy after stroke, but this thorough approach resulted in a relatively small sample size.

## 5. Conclusions

Due to the excessive constraint time and inconsistent clinical effects within different phases of stroke recovery, constraint-induced movement therapy was uncommonly put into use. Despite efforts to simplify the training programs, these programs are still questioned as to their curative effect. With consideration of therapeutic effect and clinical feasibility, the present meta-analysis identified that 6 h of training per week with 6 h constraint was efficient. However, it is recommended that the effects of this protocol be confirmed by a large scale well-designed randomized controlled trial in the future.

## Figures and Tables

**Table 1:** Characteristics of Included studies.

Authors	Years	Design	Country	Patient Number		Age (Mean)	Male (%)	Ischemic/Hemorrhagic Stroke		Lesion Side (Right/Left)		Side of Hemiparesis (Right/Left)		Handedness (Right/Left)		Post-Stroke Duration (Right/Left)		MMSE	Length of Therapy (week)	Intervention Intensity	Constraint Time (hour)	Assessment Times (week)	Outcome Measures
				CIMT	Control			CIMT	Control	CIMT	Control	CIMT	Control	CIMT	Control	CIMT	Control						
Rocha et al. [10]	2021	RCT	Brazil	15	15	59.73	70%	15/0	15/0	8/7	9/6	N/A		15/0	15/0	42.78	N/A	≥25	8	1 h/d, 3 d/wk	0	0, 4, 8	FMA-UL, MAS, SS-QOL, FRT
Baldwin et al. [11]	2018	RCT	Australia	10	9	59.25	68%	N/A		N/A		4/6	3/6	N/A		6.76	N/A	≥25	2	1 h/d, 3 d/wk	12	0, 2, 6	MAL, WMFT
Smania et al. [17]	2012	RCT	Italian	30	29	66.05	83%	25/5	25/4	N/A		14/16	13/16	28/2	28/1	10.25	N/A	≥24	2	2 h/d, 5 d/wk	12	0, 2, 14	MAL, WMFT, AS
Brunner et al. [38]	2012	RCT	Norway	14	16	63	63%	13/1	12/4	N/A		6/8	6/10	N/A		1.41	N/A	≥24	4	4 h/d, 1 d/wk	4	0, 4, 16	ARAT, 9HPT, MAL
Tariah et al. [39]	2010	RCT	Jordon	10	8	57.38	67%	N/A		7/3	4/4	N/A		N/A		9.38	CIMT: 8; CG: 9.63	≥24	8	2 h/d, 7 d/wk	2	0, 8, 24	WMFT, MAL, FMA
Lin et al. [40]	2010	RCT	Taiwan	5	8	49.6	85%	N/A		1/4	5/3	N/A		N/A		18.3	N/A	≥24	3	2 h/d, 5 d/wk	6	0, 3	MAL, FMA-UL

ARAT, Action Research Arm Test; AS, Ashworth scale; CIMT, constraint-induced movement therapy; FRT, Functional Reach Test; MMSE, mini-mental state examination; MAL, Motor Activity Log; MAS, Modified Ashworth scale; 9HPT, Nine-Hole Peg Test; SS-QOL, Stroke Specific Quality of Life; WMFT, Wolf Motor Function Test; FMA, Fugl-Meyer assessment; UL, upper limb. N/A: Not available.

**Table 2:** Meta-regression analysis.

Outcome Measure	Variables	Study Number (*n*)	Coefficient (95%CI)	*p* Value
MAL (AOU)	Post-stroke duration	5	0.113 (0.037 to 0.189)	0.0035
	Intervention time	4	0.013 (−0.005 to 0.031)	0.1670
	Constraint time	5	0.157 (0.07 to 0.243)	0.0004
	Hours a week	5	0.118 (0.022 to 0.214)	0.0159
	Course	5	−0.002 (−0.023 to 0.019)	0.8526
	Total training time	5	−0.003 (−0.013 to 0.008)	0.6135
MAL (QOM)	Post-stroke duration	5	0.115 (0.039 to 0.190)	0.0031
	Intervention time	4	0.010 (−0.009 to 0.028)	0.3017
	Constraint time	5	0.133 (0.047 to 0.219)	0.0024
	Hours a week	5	0.122 (0.026 to 0.219)	0.0126
	Course	5	0.004 (−0.017 to 0.025)	0.7243
	Total training time	5	0.000 (−0.011 to 0.011)	0.9922
WMFT (FA)	Post-stroke duration	3	0.111 (−0.116 to 0.338)	0.3375
	Intervention time	3	0.004 (−0.008 to 0.017)	0.5063
	Constraint time	3	0.026 (−0.037 to 0.088)	0.4202
	Hours a week	3	0.003 (−0.073 to 0.080)	0.9329
	Course	3	−0.005 (−0.018 to 0.008)	0.4607
	Total training time	3	−0.002 (−0.009 to 0.004)	0.4922
FMA	Post-stroke duration	3	−0.214 (−0.456 to 0.027)	0.0818
	Intervention time	3	0.121 (−0.002 to 0.244)	0.0548
	Constraint time	3	1.656 (−0.014 to 3.326)	0.0520
	Hours a week	3	0.628 (−0.118 to 1.374)	0.0992
	Course	3	0.044 (−0.181 to 0.269)	0.7028
	Total training time	3	0.041 (−0.050 to 0.133)	0.3783

MAL, Motor Activity Log; AOU, amount of use; QOM, quality of movement; WMFT, Wolf Motor Function Test; FA, functional ability; FMA, Fugl-Meyer assessment; CI, confidence interval.

## Data Availability

All data accessed and analyzed in this study are available in the article.

## Supplementary Materials

The following supporting information can be downloaded at: https://www.mdpi.com/article/10.3390/jcm12062267/s1, Table S1: Search strategy; Figure S1A: Risk of bias graph and figure; Figure S1B: Risk of bias summary; Figure S2: Subgroup analysis of outcomes.
