# Supplementary material for: Timing and Dose of Constraint-Induced Movement Therapy after Stroke: A Systematic Review and Meta-Regression
Source: J Clin Med. 2023 Mar 15;12(6):2267. doi: 10.3390/jcm12062267 (PMC10058952; doi:10.3390/jcm12062267)
Supplement: Supplementary file 1 [file jcm-12-02267-s001.zip › jcm-2178988-supplementary.pdf]

Supplementary Materials

Efficacy of constraint-induced movement therapy in stroke patients with fair cognitive function: A systematic review and meta-regression

Yu-Kai Yang MD, Chieh-Yu Lin MD, Po-Huang Chen MD, Hong-Jie Jhou MD

Contents

Supplementary Materials S1. PRISMA Checklist

Supplementary Materials S2. PRISMA flow diagram

Supplementary Materials S3. Search strategy

Supplementary Materials S4. Reference list of full-text screening studies

Supplementary Materials S5. Assessment of risk of bias

Supplementary Materials S1. PRIMSA checklist

Section and Topic	Item #	Checklist item	Location where item is reported
**TITLE**			
Title	1	Identify the report as a systematic review.	1
**ABSTRACT**			
Abstract	2	See the PRISMA 2020 for Abstracts checklist.	1
**INTRODUCTION**			
Rationale	3	Describe the rationale for the review in the context of existing knowledge.	1-2
Objectives	4	Provide an explicit statement of the objective(s) or question(s) the review addresses.	1-2
**METHODS**			
Eligibility criteria	5	Specify the inclusion and exclusion criteria for the review and how studies were grouped for the syntheses.	2
Information sources	6	Specify all databases, registers, websites, organisations, reference lists and other sources searched or consulted to identify studies. Specify the date when each source was last searched or consulted.	2
Search strategy	7	Present the full search strategies for all databases, registers and websites, including any filters and limits used.	2
Selection process	8	Specify the methods used to decide whether a study met the inclusion criteria of the review, including how many reviewers screened each record and each report retrieved, whether they worked independently, and if applicable, details of automation tools used in the process.	2-3
Data collection process	9	Specify the methods used to collect data from reports, including how many reviewers collected data from each report, whether they worked independently, any processes for obtaining or confirming data from study investigators, and if applicable, details of automation tools used in the process.	3

Section and Topic	Item #	Checklist item	Location where item is reported
Data items	10a	List and define all outcomes for which data were sought. Specify whether all results that were compatible with each outcome domain in each study were sought (e.g. for all measures, time points, analyses), and if not, the methods used to decide which results to collect.	3
	10b	List and define all other variables for which data were sought (e.g. participant and intervention characteristics, funding sources). Describe any assumptions made about any missing or unclear information.	3
Study risk of bias assessment	11	Specify the methods used to assess risk of bias in the included studies, including details of the tool(s) used, how many reviewers assessed each study and whether they worked independently, and if applicable, details of automation tools used in the process.	3, Figure S1
Effect measures	12	Specify for each outcome the effect measure(s) (e.g. risk ratio, mean difference) used in the synthesis or presentation of results.	3-4
Synthesis methods	13a	Describe the processes used to decide which studies were eligible for each synthesis (e.g. tabulating the study intervention characteristics and comparing against the planned groups for each synthesis (item #5)).	3-4
	13b	Describe any methods required to prepare the data for presentation or synthesis, such as handling of missing summary statistics, or data conversions.	3-4
	13c	Describe any methods used to tabulate or visually display results of individual studies and syntheses.	3-4
	13d	Describe any methods used to synthesize results and provide a rationale for the choice(s). If meta-analysis was performed, describe the model(s), method(s) to identify the presence and extent of statistical heterogeneity, and software package(s) used.	4
	13e	Describe any methods used to explore possible causes of heterogeneity among study results (e.g. subgroup analysis, meta-regression).	3-4
	13f	Describe any sensitivity analyses conducted to assess robustness of the synthesized results.	Sensitivity analysis was not conducted in the study

Section and Topic	Item #	Checklist item	Location where item is reported
Reporting bias assessment	14	Describe any methods used to assess risk of bias due to missing results in a synthesis (arising from reporting biases).	3
Certainty assessment	15	Describe any methods used to assess certainty (or confidence) in the body of evidence for an outcome.	3-4
**RESULTS**			
Study selection	16a	Describe the results of the search and selection process, from the number of records identified in the search to the number of studies included in the review, ideally using a flow diagram.	4-5, Figure1
	16b	Cite studies that might appear to meet the inclusion criteria, but which were excluded, and explain why they were excluded.	4-5, Figure1
Study characteristics	17	Cite each included study and present its characteristics.	5, Table1
Risk of bias in studies	18	Present assessments of risk of bias for each included study.	S1A, S1B
Results of individual studies	19	For all outcomes, present, for each study: (a) summary statistics for each group (where appropriate) and (b) an effect estimate and its precision (e.g. confidence/credible interval), ideally using structured tables or plots.	6-7, Figure2 and 3
Results of syntheses	20a	For each synthesis, briefly summarise the characteristics and risk of bias among contributing studies.	7-10
	20b	Present results of all statistical syntheses conducted. If meta-analysis was done, present for each the summary estimate and its precision (e.g. confidence/credible interval) and measures of statistical heterogeneity. If comparing groups, describe the direction of the effect.	7-10, table2, Figure4, S2
	20c	Present results of all investigations of possible causes of heterogeneity among study results.	7-10
	20d	Present results of all sensitivity analyses conducted to assess the robustness of the synthesized results.	Sensitivity analysis

Section and Topic	Item #	Checklist item	Location where item is reported
			was not conducted in the study
Reporting biases	21	Present assessments of risk of bias due to missing results (arising from reporting biases) for each synthesis assessed.	7-10
Certainty of evidence	22	Present assessments of certainty (or confidence) in the body of evidence for each outcome assessed.	7-10
**DISCUSSION**			
Discussion	23a	Provide a general interpretation of the results in the context of other evidence.	10
	23b	Discuss any limitations of the evidence included in the review.	11
	23c	Discuss any limitations of the review processes used.	11
	23d	Discuss implications of the results for practice, policy, and future research.	11
**OTHER INFORMATION**			
Registration and protocol	24a	Provide registration information for the review, including register name and registration number, or state that the review was not registered.	2
	24b	Indicate where the review protocol can be accessed, or state that a protocol was not prepared.	2
	24c	Describe and explain any amendments to information provided at registration or in the protocol.	There is no amendment in this protocol.
Support	25	Describe sources of financial or non-financial support for the review, and the role of the funders or sponsors in the review.	11

Section and Topic	Item #	Checklist item	Location where item is reported
Competing interests	26	Declare any competing interests of review authors.	11
Availability of data, code and other materials	27	Report which of the following are publicly available and where they can be found: template data collection forms; data extracted from included studies; data used for all analyses; analytic code; any other materials used in the review.	3, Table 1
	23d	Discuss implications of the results for practice, policy, and future research.	11
**OTHER INFORMATION**			
Registration and protocol	24a	Provide registration information for the review, including register name and registration number, or state that the review was not registered.	2
	24b	Indicate where the review protocol can be accessed, or state that a protocol was not prepared.	2
	24c	Describe and explain any amendments to information provided at registration or in the protocol.	There is no amendment in this protocol.
Support	25	Describe sources of financial or non-financial support for the review, and the role of the funders or sponsors in the review.	11
Competing interests	26	Declare any competing interests of review authors.	11
Availability of data, code and other materials	27	Report which of the following are publicly available and where they can be found: template data collection forms; data extracted from included studies; data used for all analyses; analytic code; any other materials used in the review.	3, Table 1

Supplementary Materials S2. PRISMA flow diagram

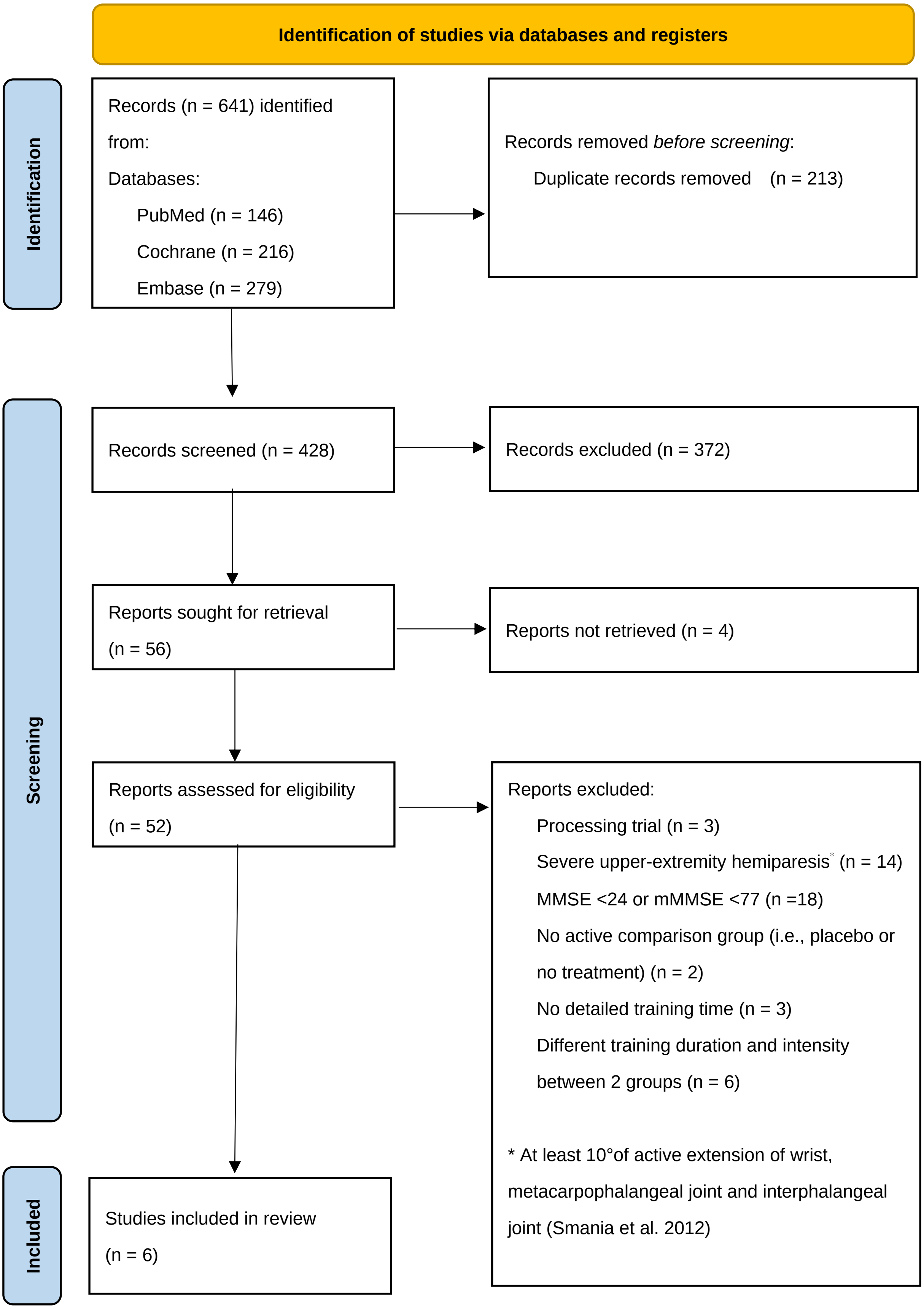

Supplementary Materials S3. Search Strategy

Table S1: Search strategy

	Key words	Synonyms
**Population**	Patient with first-time stroke	Patient with first-time stroke
**Intervention**	constraint-induced movement therapy	constraint-induced movement therapy, forced-use therapy
**Control**	Tradiional rehabilitation therapy	Usual care, standard care
**Outcome**	Fugl-Meyer assessment (FMA), Motor Activity Log (MAL), Wolf Motor Function Test (WMFT)	Fugl-Meyer assessment (FMA), Motor Activity Log (MAL), Wolf Motor Function Test (WMFT)

Pubmed

#1 (stroke OR infarction)

#2 (“forced use” OR “constraint induced movement therapy” OR “constraint induced therapy”)

#3 1 AND 2

#4 1 AND #2 AND” randomized controlled trial”

Embase

#1 'stroke'/exp OR stroke OR 'infarction'/exp OR infarction

#2 forced AND use OR (constraint AND induced AND movement AND therapy) OR (constraint AND induced AND therapy)

#1 AND #2

#1 AND #2 AND 'randomized controlled trial'/de

Cochrane Library

#1 (stroke OR infarction)

#2 (“forced use” OR “constraint induced movement therapy” OR “constraint induced therapy”)

#3 1 AND 2

#4 1 AND #2 AND” randomized controlled trial”

Supplementary Materials S4. Reference list of full-text screening studies

Included studies **(No. 1 to No. 6)**

1. Rocha, L., et al., *Constraint Induced Movement Therapy Increases Functionality and Quality of Life after Stroke.* Journal of Stroke and Cerebrovascular Diseases, 2021, 30(6) : 105774
2. Baldwin, C., et al., *Modified Constraint-Induced Movement Therapy is a feasible and potentially useful addition to the Community Rehabilitation tool kit after stroke: A pilot randomised control trial.* Australian occupational therapy journal, 2018, 65(6), p. 503–511.
3. Smania, N., et al., *Reduced-intensity modified constraint-induced movement therapy versus conventional therapy for upper extremity rehabilitation after stroke: a multicenter trial.* Neurorehabilitation and neural repair, 2012, 26(9), p. 1035–1045.
4. Brunner, I. C., et al., *Is modified constraint-induced movement therapy more effective than bimanual training in improving arm motor function in the subacute phase post stroke? A randomized controlled trial.* Clinical rehabilitation, 2012, 26(12), p. 1078–1086.
5. Tariah et al., *Constraint induced movement therapy for stroke survivors in Jordon: a home-based model.* International Journal of Therapy and Rehabilitation, 2010, 17(12)
6. Lin, K. C., et al., *Constraint-induced therapy versus control intervention in patients with stroke: a functional magnetic resonance imaging study.* American journal of physical medicine & rehabilitation, 2010, 89(3), p. 177–185.
7. Waris M., et al., *Comparison of constraint-induced movement therapy vs traditional rehabilitation therapy to improve upper limb function in hemiplegic acute and subacute stroke patients.* Pakistan Journal of Medical and Health Sciences, 2021, 15:10, p. 3021-3025

8. Kale AA., et al., *Effect of Constraint Induced Movement Therapy Versus Bimanual Task Training for Improvement of Motor Hand Function in Stroke Patients.* Indian journal of physiotherapy & occupational therapy, 2019, 13(1), p. 23-27
9. Abdullahi A., et al., *Effects of Number of Repetitions and Number of Hours of Shaping Practice during Constraint-Induced Movement Therapy: A Randomized Controlled Trial.* Neurology research international, 2018, 5496408.
10. Gauthier, L. V., et al., *Video Game Rehabilitation for Outpatient Stroke (VIGoROUS): protocol for a multi-center comparative effectiveness trial of in-home gamified constraint-induced movement therapy for rehabilitation of chronic upper extremity hemiparesis.* BMC neurology, 2017, 17(1), 109.
11. Yu, C., et al., *The Effects of Modified Constraint-Induced Movement Therapy in Acute Subcortical Cerebral Infarction.* Frontiers in human neuroscience, 2017, 11, 265.
12. Liu, K. P., et al., *A randomized controlled trial of self-regulated modified constraint-induced movement therapy in sub-acute stroke patients.* European journal of neurology, 2016, 23(8), p. 1351–1360.
13. Yadav, R. K., et al., *Efficacy of Modified Constraint Induced Movement Therapy in the Treatment of Hemiparetic Upper Limb in Stroke Patients: A Randomized Controlled Trial.* Journal of clinical and diagnostic research: JCDR, 2016, 10(11), YC01–YC05.

14. Kwakkel, G.,et al., *Effects of Unilateral Upper Limb Training in Two Distinct Prognostic Groups Early After Stroke: The EXPLICIT-Stroke Randomized Clinical Trial.* Neurorehabilitation and neural repair, 2016, 30(9), p. 804–816.
15. Barzel, A., et al., *Home-based constraint-induced movement therapy for patients with upper limb dysfunction after stroke (HOMECIMT): a cluster-randomised, controlled trial.* The Lancet. Neurology, 2015, 14(9), p. 893–902.
16. van Delden, et al., *Unilateral and bilateral upper-limb training interventions after stroke have similar effects on bimanual coupling strength.* Neurorehabilitation and neural repair, 2015, 29(3), 255–267.
17. Yoon, J. A., et al. *Effect of constraint-induced movement therapy and mirror therapy for patients with subacute stroke.* Annals of rehabilitation medicine, 2014, 38(4), p. 458–466.
18. Singh, P., et al., *Study to assess the effectiveness of modified constraint-induced movement therapy in stroke subjects: A randomized controlled trial.* Annals of Indian Academy of Neurology, 2013, 16(2), p. 180–184.
19. van Delden, A. L., et al., *Unilateral versus bilateral upper limb training after stroke: the Upper Limb Training After Stroke clinical trial.* Stroke, 2013, 44(9), p. 2613–2616.
20. Wu, C. Y., et al., *Constraint-induced therapy with trunk restraint for improving functional outcomes and trunk-arm control after stroke: a randomized controlled trial.* Physical therapy, 2012, 92(4), p. 483–492.
21. Kitago, T., et al. *Improvement after constraint-induced movement therapy: recovery of normal motor control or task-specific compensation?.* Neurorehabilitation and neural repair, 2013, 27(2), 99–109.
22. Khan, C. M., et al., *Potential effectiveness of three different treatment approaches to improve minimal to moderate arm and hand function after stroke--a pilot randomized clinical trial.* Clinical rehabilitation, 2011, 25(11), p. 1032–1041.
23. Wu, C. Y., et al., *Randomized trial of distributed constraint-induced therapy versus bilateral arm training for the rehabilitation of upper-limb motor control and function after stroke.* Neurorehabilitation and neural repair, 2011, 25(2), p. 130–139.
24. Wang, Q., *Comparison of conventional therapy, intensive therapy and modified constraint-induced movement therapy to improve upper extremity function after stroke.* Journal of rehabilitation medicine, 2011, 43(7), p. 619–625.
25. Hayner, K., et al., *Comparison of constraint-induced movement therapy and bilateral treatment of equal intensity in people with chronic upper-extremity dysfunction after cerebrovascular accident.* The American journal of occupational therapy: official publication of the American Occupational Therapy Association, 2010, 64(4), p. 528–539.
26. van Delden, A. L., et al., *Comparing unilateral and bilateral upper limb training: the ULTRA-stroke program design.* BMC neurology, 2009, 9, 57.
27. Dromerick, A. W., et al., *Very Early Constraint-Induced Movement during Stroke Rehabilitation (VECTORS): A single-center RCT.* Neurology, 2009, 73(3), p. 195–201.

28. Hammer, A. M., et al., *Effects of forced use on arm function in the subacute phase after stroke: a randomized, clinical pilot study.* Physical therapy, 2009, 89(6), p. 526–539.

29. Lin, K. C., et al., *Effects of constraint-induced therapy versus bilateral arm training on motor performance, daily functions, and quality of life in stroke survivors.* Neurorehabilitation and neural repair, 2009, 23(5), p. 441–448.

30. Lin, K. C., et al., *Constraint-induced therapy versus dose-matched control intervention to improve motor ability, basic/extended daily functions, and quality of life in stroke.* Neurorehabilitation and neural repair, 2009, 23(2), p. 160–165.

31. Myint, M. W., et al., *Use of constraint-induced movement therapy in Chinese stroke patients during the sub-acute period.* Hong Kong medical journal, 2009, 14(5 Suppl), p. 40–42.

32. Sawaki, L., et al., *Constraint-induced movement therapy results in increased motor map area in subjects 3 to 9 months after stroke.* Neurorehabilitation and neural repair, 2009, 22(5), p. 505–513.

33. Massie, C., et al. *The effects of constraint-induced therapy on kinematic outcomes and compensatory movement patterns: an exploratory study.* Archives of physical medicine and rehabilitation, 2009, 90(4), 571–579.

34. Lin, K. C., et al., A randomized controlled trial of constraint-induced movement therapy after stroke. Acta neurochirurgica. 2008, Supplement, 101, p. 61–64.

35. Kwakkel, G., et al. *Impact of early applied upper limb stimulation: the EXPLICIT-stroke programme design.* BMC neurology, 2008, 8, 49.

36. Caimmi, M., et al. *Using kinematic analysis to evaluate constraint-induced movement therapy in chronic stroke patients. Neurorehabilitation and neural repair,* 2008, 22(1), 31–39.

37. Kim, D. G., et al., *Effect of constraint-induced movement therapy with modified opposition restriction orthosis in chronic hemiparetic patients with stroke.* NeuroRehabilitation, 2008, 23(3), p. 239–244.

38. Dahl, A. E., et al., *Short- and long-term outcome of constraint-induced movement therapy after stroke: a randomized controlled feasibility trial.* Clinical rehabilitation, 2008, 22(5), p. 436–447.

39. Page, S. J., et al., *Modified constraint-induced therapy in chronic stroke: results of a single-blinded randomized controlled trial.* Physical therapy, 2008, 88(3), p. 333–340.

40. Wolf, S. L., et al., *Retention of upper limb function in stroke survivors who have received constraint-induced movement therapy: the EXCITE randomised trial.* The Lancet. Neurology, 2008, 7(1), 33–40.

41. Lin, K. C., et al., *Effects of modified constraint-induced movement therapy on reach-to-grasp movements and functional performance after chronic stroke: a randomized controlled study.* Clinical rehabilitation, 2007, 21(12), p. 1075–1086.

42. Wu, C. Y., et al., *Kinematic and clinical analyses of upper-extremity movements after constraint-induced movement therapy in patients with stroke: a randomized controlled trial.* Archives of physical medicine and rehabilitation, 2007, 88(8), p. 964–970.

43. Wu, C. Y.,et al., *Effects of modified constraint-induced movement therapy on movement kinematics and daily function in patients with stroke: a kinematic study of motor control mechanisms.* Neurorehabilitation and neural repair, 2007, 21(5), p. 460–466.

44. Boake, C., et al., *Constraint-induced movement therapy during early stroke rehabilitation.* Neurorehabilitation and neural repair, 2007, 21(1), p. 14–24.

45. Ro, T., Noser, et al., *Functional reorganization and recovery after constraint-induced movement therapy in subacute stroke: case reports.* Neurocase, 2006, 12(1), p. 50–60.

46. Wolf, S. L., et al., *Effect of constraint-induced movement therapy on upper extremity function 3 to 9 months after stroke: the EXCITE randomized clinical trial.* JAMA, 2006, 296(17), p. 2095–2104.

47. Yen, J. G., et al., *Effectiveness of modified constraint-induced movement therapy on upper limb function in stroke subjects.* Acta neurologica Taiwanica, 2005, 14(1), p. 16–20.

48. Page, S. J., et al., *Modified constraint-induced therapy in acute stroke: a randomized controlled pilot study.* Neurorehabilitation and neural repair, 2005, 19(1), p. 27–32.

49. Suputtitada, A., et al. *Effectiveness of constraint-induced movement therapy in chronic stroke patients.* Journal of the Medical Association of Thailand = Chotmaihet thangphaet, 2004, 87(12), p. 1482–1490.

50. Ploughman, M., et al., *Can forced-use therapy be clinically applied after stroke? An exploratory randomized controlled trial.* Archives of physical medicine and rehabilitation, 2004, 85(9), p. 1417–1423.

51. Page, S. J., et al., *Efficacy of modified constraint-induced movement therapy in chronic stroke: a single-blinded randomized controlled trial.* Archives of physical medicine and rehabilitation, 2004, 85(1), p. 14–18.

52. Alberts, J. L., et al., *The effects of constraint-induced therapy on precision grip: a preliminary study.* Neurorehabilitation and neural repair, 2004, 18(4), p. 250–258.

53. Wittenberg, G. F., et al., *Constraint-induced therapy in stroke: magnetic-stimulation motor maps and cerebral activation.* Neurorehabilitation and neural repair, 2003, 17(1), p. 48–57.

54. Page, S. J., et al., *Modified constraint-induced therapy after subacute stroke: a preliminary study.* Neurorehabilitation and neural repair, 2002, 16(3), p. 290–295.

55. Page, S. J., et al., *Modified constraint induced therapy: a randomized feasibility and efficacy study.* Journal of rehabilitation research and development, 2001, 38(5), p. 583–590.

56. Dromerick, A. W., et al., *Does the application of constraint-induced movement therapy during acute rehabilitation reduce arm impairment after ischemic stroke?* Stroke, 2000, 31(12), p. 2984–2988.

Supplementary Materials S5. Assessment of risk of bias

Figure S1A: Risk of bias graph and figure

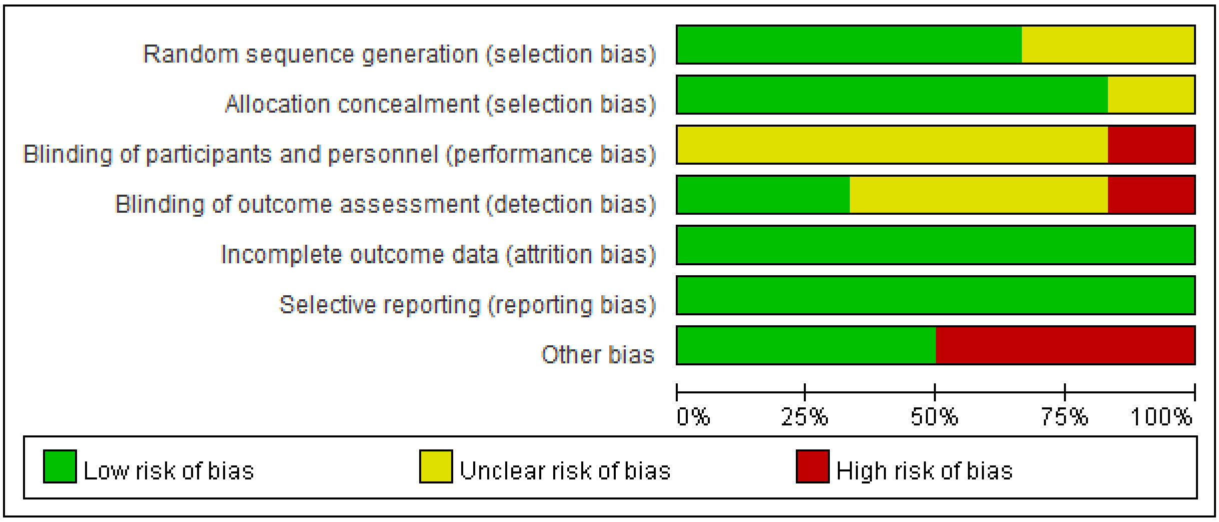

Figure S1B: Risk of bias summary

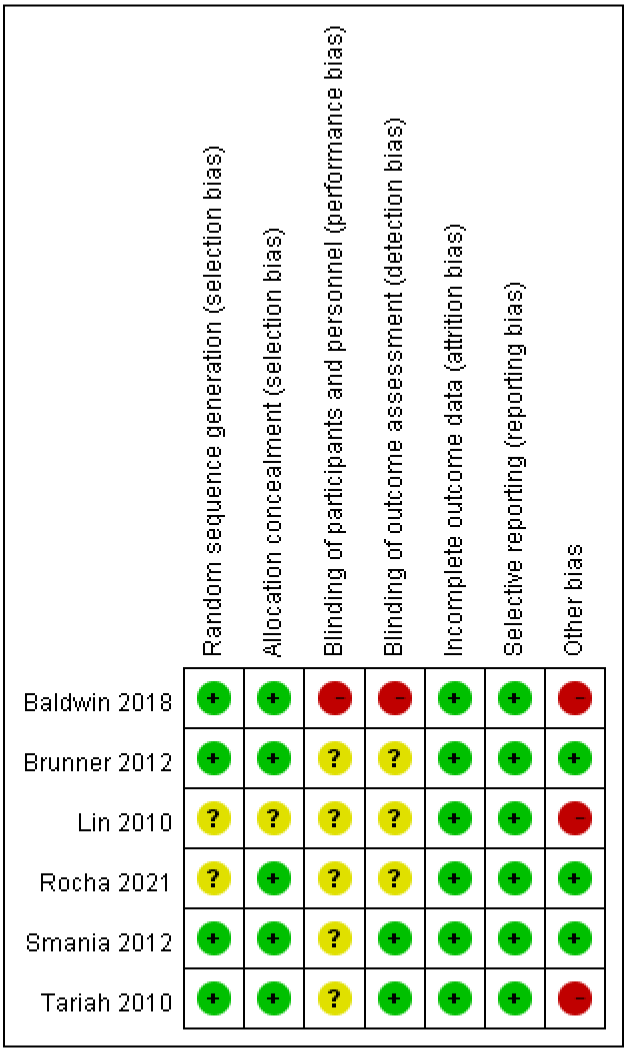

Figure S2. Subgroup analysis of outcomes

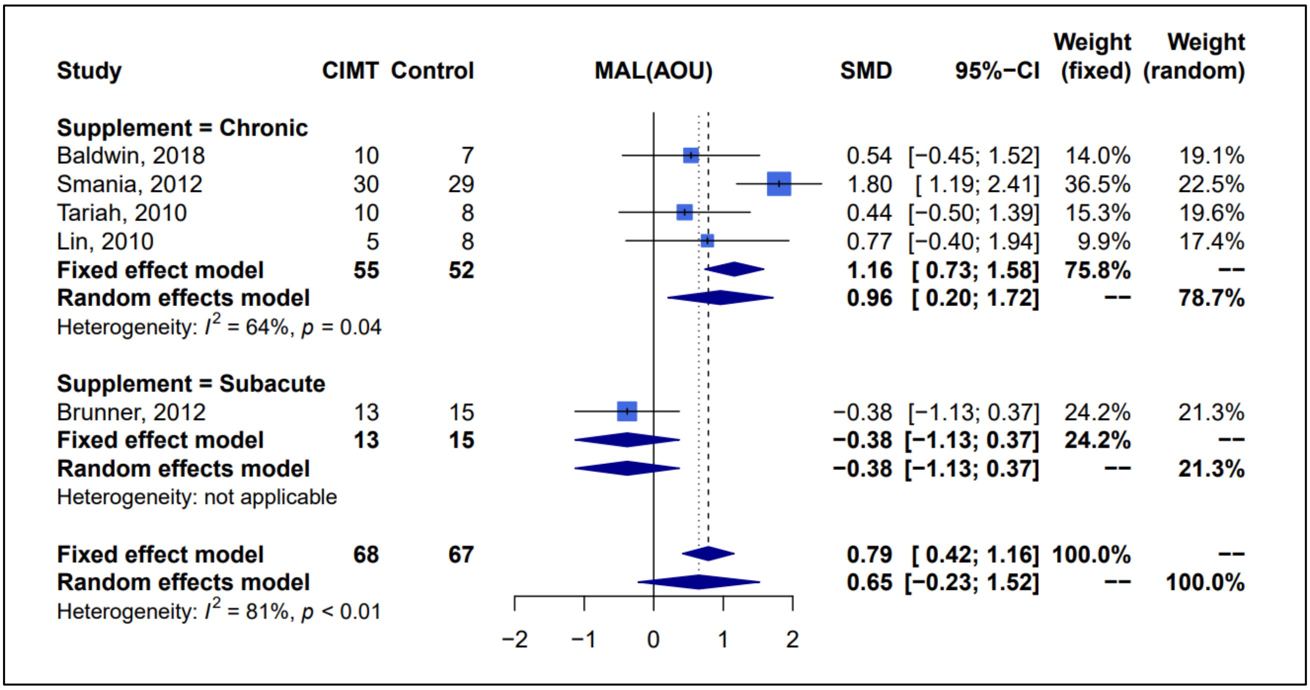

Subgroup analysis of different post stroke duration in outcome of MAL-AOU

The included patients were categorized by chronic or subacute status. Outcome analyses were performed using standardised mean difference with related 95% confidence intervals (95%CI). CIMT, Constraint-induced movement therapy; MAL, Motor Activity Log; AOU, amount of use; SMD, standardised mean difference; CI, confidence interval

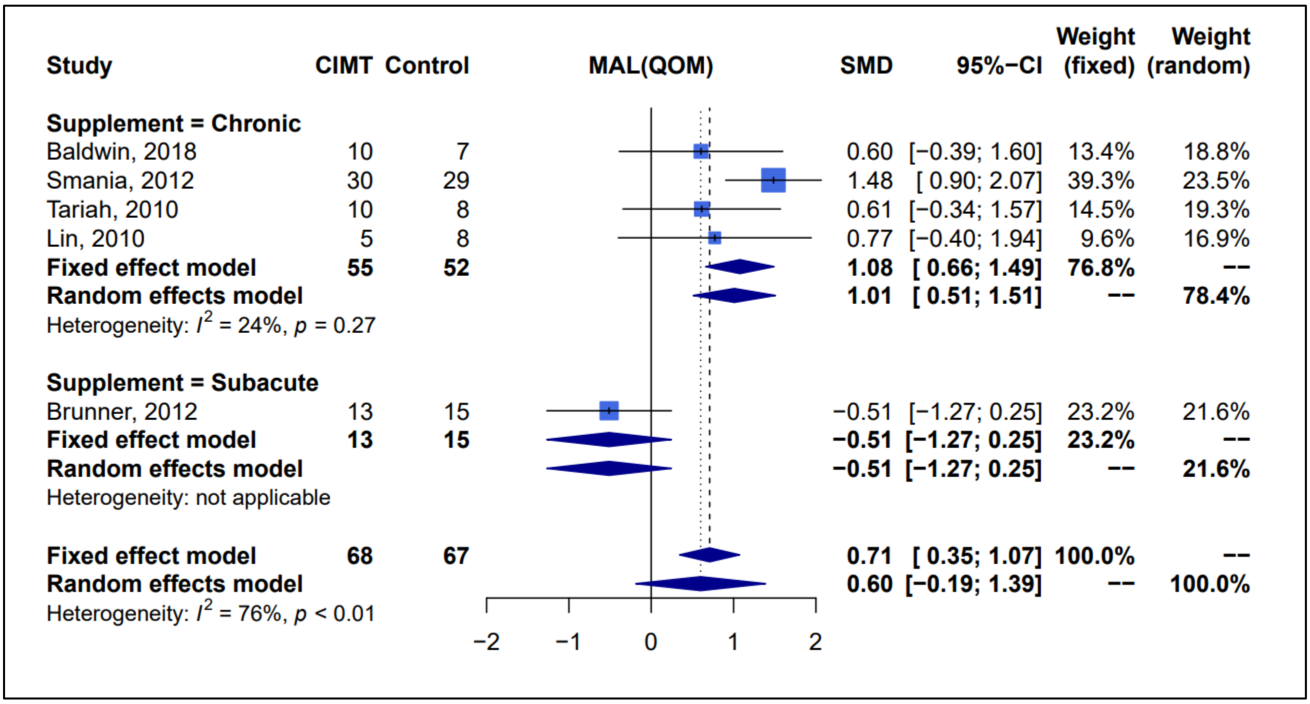

Subgroup analysis of different post stroke duration in outcome of MAL-QOM

The included patients were categorized by chronic or subacute status. Outcome analyses were performed using standardised mean difference with related 95% confidence intervals (95%CI). CIMT, Constraint-induced movement therapy; MAL, Motor Activity Log; QOM, quality of movement; SMD, standardised mean difference; CI, confidence interval

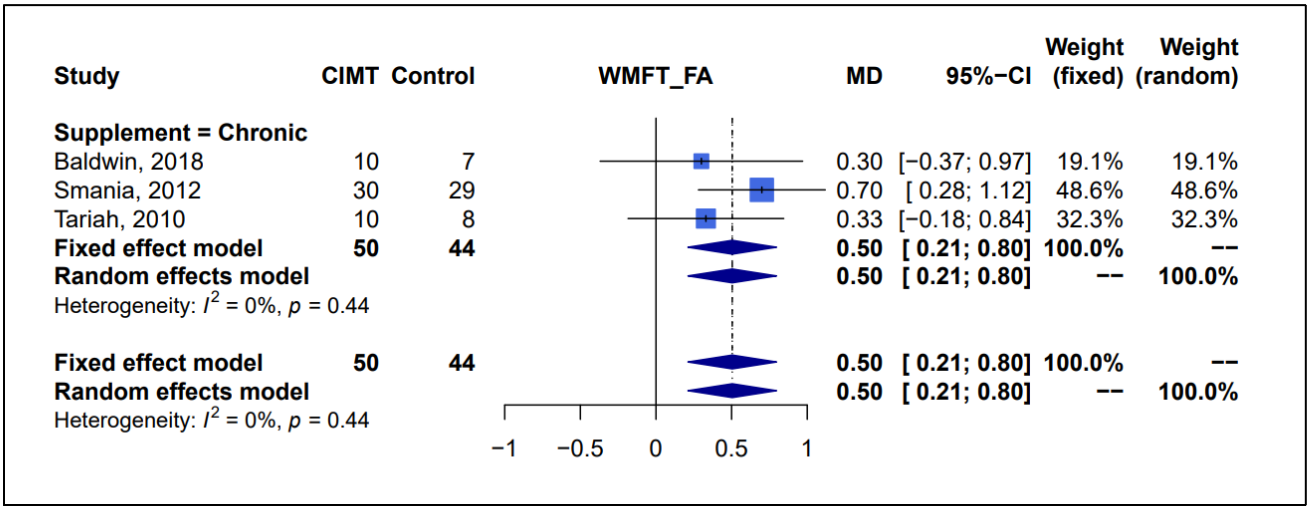

Subgroup analysis of different post stroke duration in outcome of WMFT-FA

The included patients were categorized by chronic or subacute status. Outcome analyses were performed using mean difference with related 95% confidence intervals (95%CI).

CIMT, Constraint-induced movement therapy; WMFT, Wolf Motor Function Test; FA, functional ability; MD, mean difference; CI, confidence interval

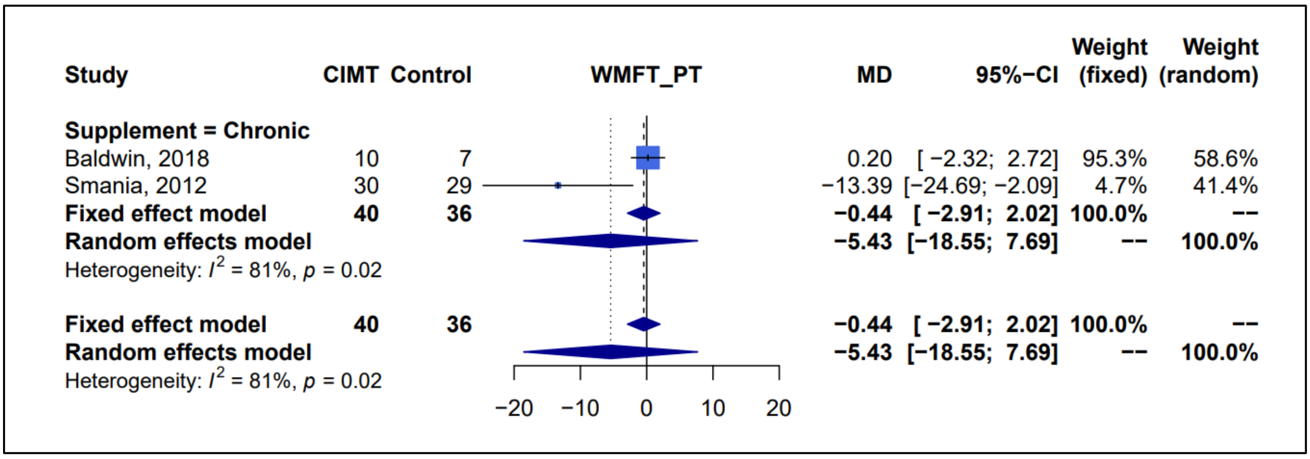

Subgroup analysis of different post stroke duration in outcome of WMFT-PT

The included patients were categorized by chronic or subacute status. Outcome analyses were performed using mean difference with related 95% confidence intervals (95%CI).

CIMT, Constraint-induced movement therapy; WMFT, Wolf Motor Function Test; PT, performance time; MD, mean difference; CI, confidence interval

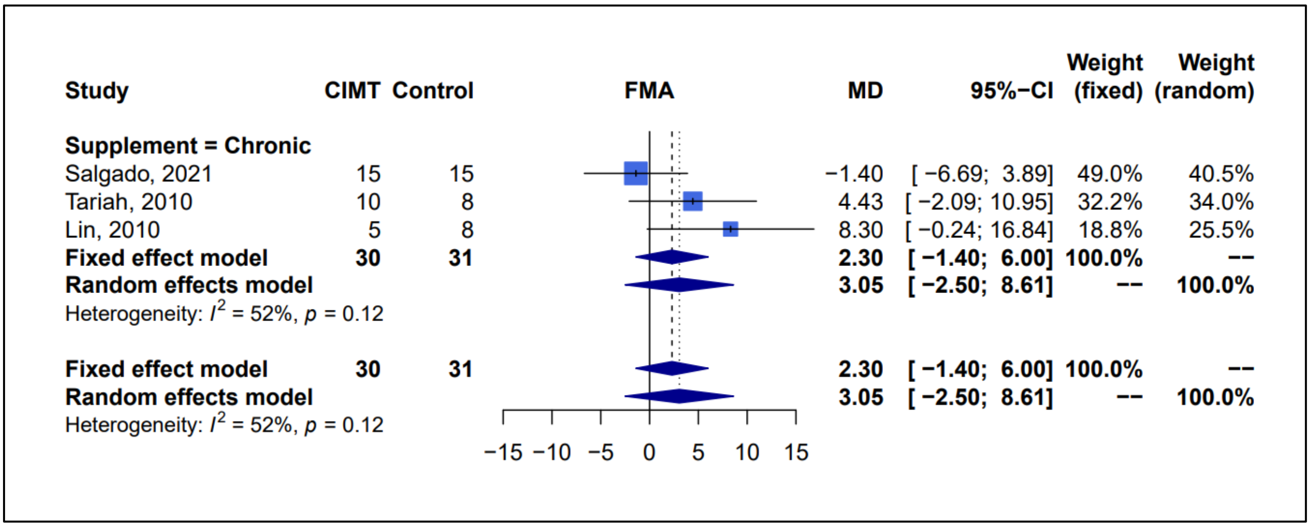

Subgroup analysis of different post stroke duration in outcome of FMA

The included patients were categorized by chronic or subacute status. Outcome analyses were performed using mean difference with related 95% confidence intervals (95%CI).

CIMT, Constraint-induced movement therapy; FMA, Fugl-Meyer assessment; MD, mean difference; CI, confidence interval

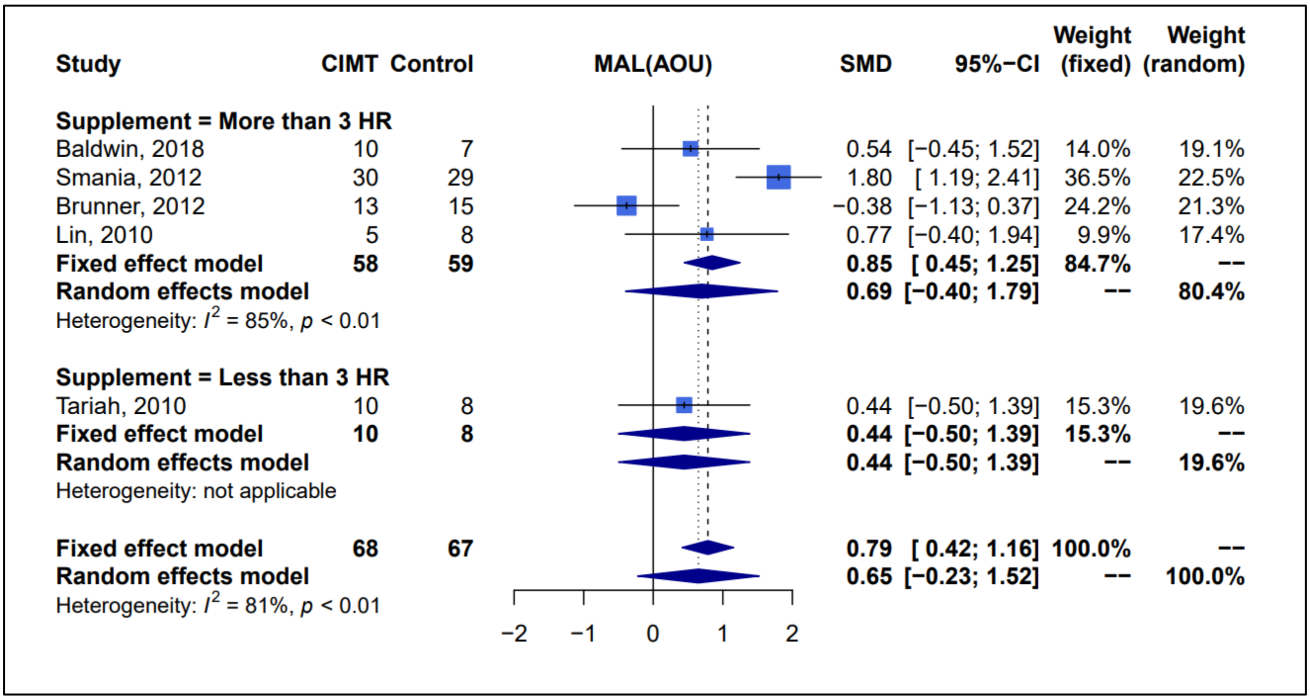

Subgroup analysis of different constriant time in outcome of MAL-AOU

The included patients were categorized by receiving more or less than 3 hours of constriant time . Outcome analyses were performed using standardised mean difference with related 95% confidence intervals (95%CI).

CIMT, Constraint-induced movement therapy; MAL, Motor Activity Log; AOU, amount of use; SMD, standardised mean difference; CI, confidence interval

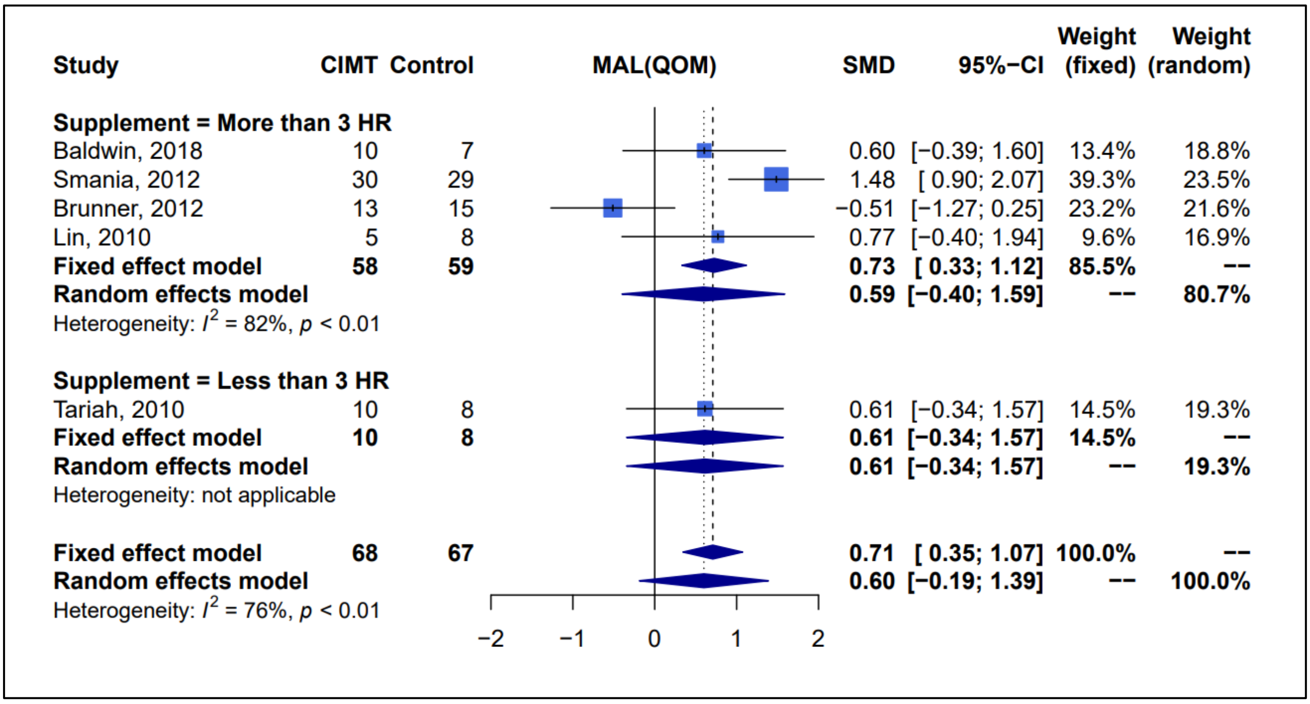

Subgroup analysis of different constriant time in outcome of MAL-QOM

The included patients were categorized by receiving more or less than 3 hours of constriant time. Outcome analyses were performed using standardised mean difference with related 95% confidence intervals (95%CI).

CIMT, Constraint-induced movement therapy; MAL, Motor Activity Log; QOM, quality of movement; SMD, standardised mean difference; CI, confidence interval

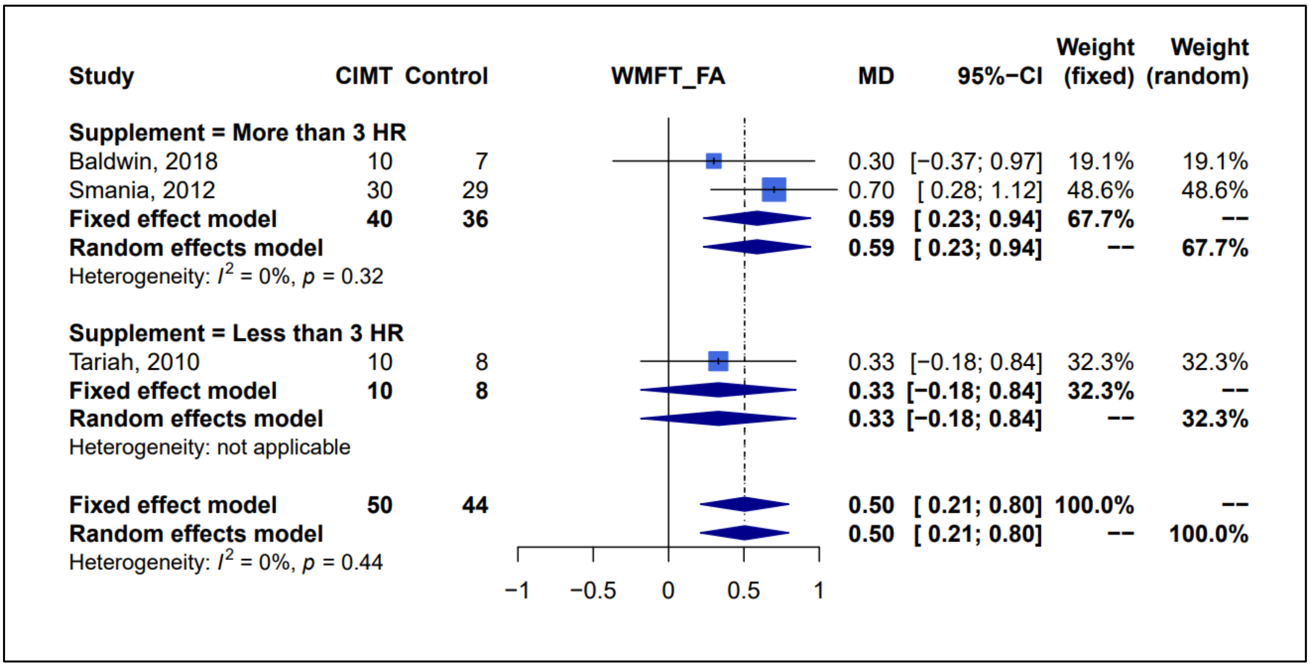

Subgroup analysis of different constriant time in outcome of WMFT-FA

The included patients were categorized by receiving more or less than 3 hours of constriant time.

Outcome analyses were performed using mean difference with related 95% confidence intervals (95%CI).

CIMT, Constraint-induced movement therapy; WMFT, Wolf Motor Function Test; FA, functional ability; MD, mean difference; CI, confidence interval

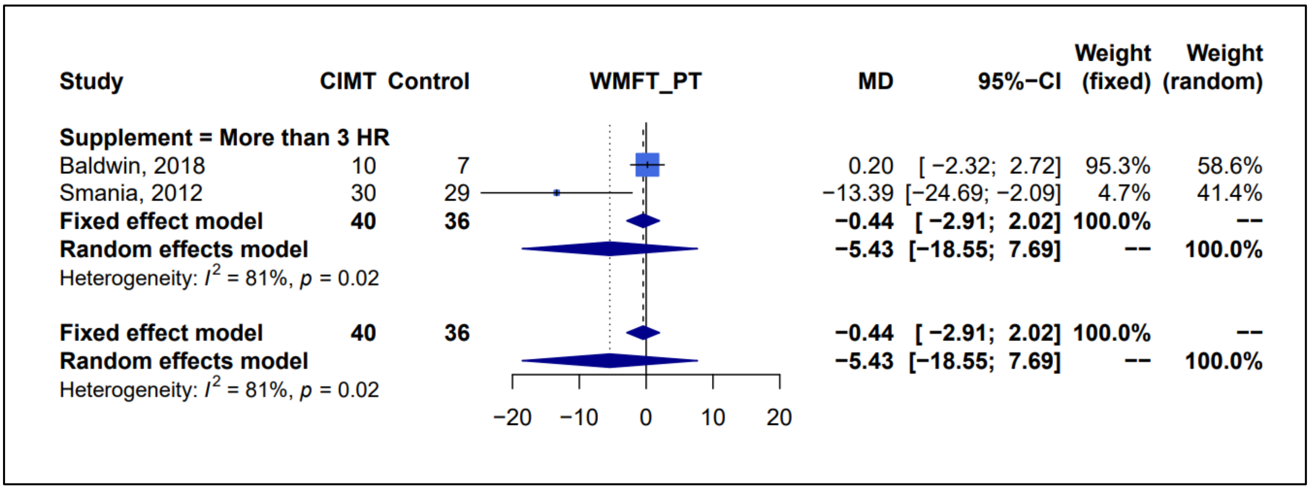

Subgroup analysis of different constriant time in outcome of WMFT-PT

The included patients were categorized by receiving more or less than 3 hours of constriant time. Outcome analyses were performed using mean difference with related 95% confidence intervals (95%CI).

CIMT, Constraint-induced movement therapy; WMFT, Wolf Motor Function Test; PT, performance time; MD, mean difference; CI, confidence interval

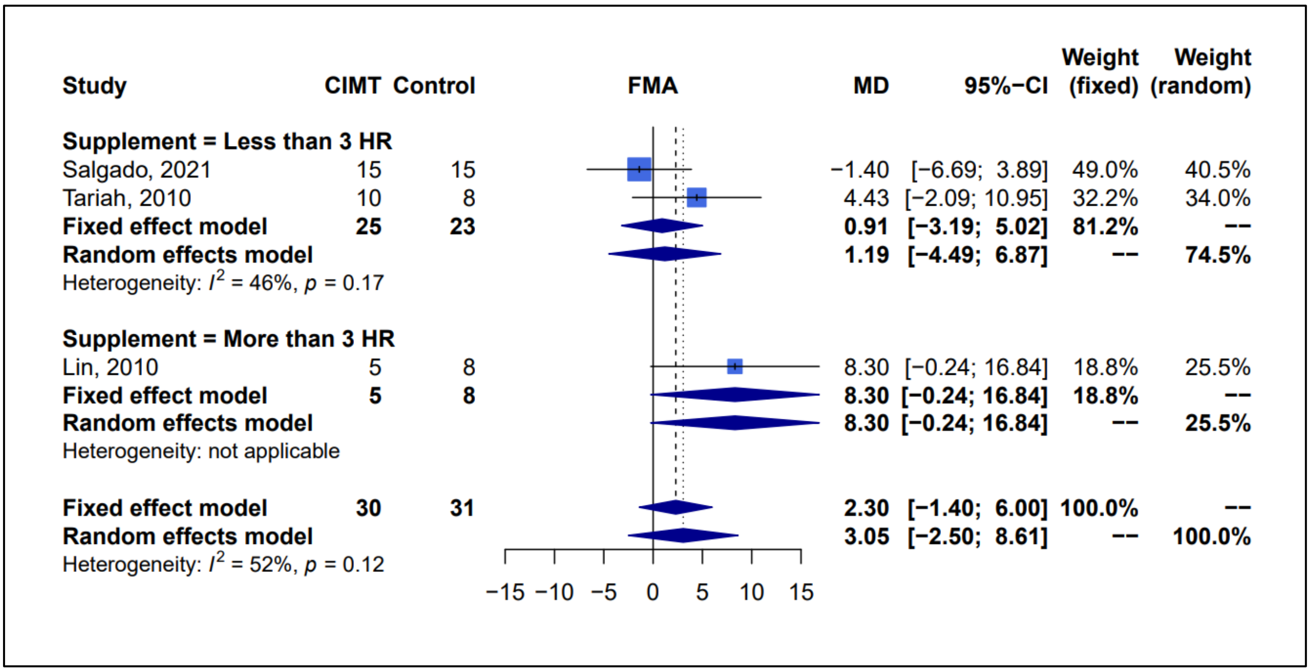

Subgroup analysis of different constriant time in outcome of FMA

The included patients were categorized by receiving more or less than 3 hours of constriant time.

Outcome analyses were performed using mean difference with related 95% confidence intervals (95%CI).

CIMT, Constraint-induced movement therapy; FMA, Fugl-Meyer assessment; MD, mean difference; CI, confidence interval

Subgroup analysis did not perform in outcomes: intervention time, hours a week, course, and total training time because both intervention group and control group received the same dose of trainging in each study.
